# Emerging frontiers in the mitochondrial regulation of dendritic cell biology

**DOI:** 10.1016/j.redox.2026.104032

**Published:** 2026-01-14

**Authors:** B. Chen, J.U. Mayer

**Affiliations:** a Department of Dermatology, University Medical Center of the Johannes Gutenberg-University Mainz, Mainz, Germany; b Research Center for Immunotherapy (FZI), University Medical Center of the Johannes Gutenberg-University Mainz, Mainz, Germany; c Institute of Quantitative & Computational Biosciences, Johannes Gutenberg University Mainz, Mainz, Germany; d Center for Healthy Ageing, Johannes Gutenberg University Mainz, Mainz, Germany

**Keywords:** Mitochondria, Dendritic cell, Mitochondrial dynamics, Mitochondrial diseases, Redox biology

## Abstract

Dendritic Cells are central players of our immune system, linking innate sensing to adaptive immunity through antigen presentation and T cell priming. Beyond transcriptional and cytokine-based regulation, mitochondria are emerging as potential regulators of Dendritic Cell biology. While still in its infancy, evidence is accumulating that mitochondrial pathways affect Dendritic Cell differentiation; that mitochondrial remodeling and bioenergetic rewiring underpin Dendritic Cell maturation and activation in response to pathogenic and inflammatory stimuli and that shifts in mitochondrial and redox dynamics, reactive oxygen species production and mitochondrial DNA release coincide with Dendritic Cell activation and co-stimulatory molecule expression. Mitochondria are furthermore involved in regulating Dendritic Cell migration by influencing cellular metabolism and cytoskeletal dynamics and support the antigen processing and presentation machinery, thereby dictating the quality of the initiated T cell response. Importantly, mitochondrial checkpoints also regulate Dendritic Cell survival, balancing immune activation with timely cell death to preserve immune homeostasis.

While the exact pathways of mitochondrial regulation are just beginning to be understood, disruptions in these programs can be far reaching. During aging, progressive mitochondrial dysfunction has been associated with impaired Dendritic Cell differentiation, diminished antigen presentation and impaired T cell responses. Similar defects have been observed in chronic diseases and cancer, leading us to hypothesize that genetic disorders linked to mitochondrial dysfunction also lead to defects in Dendritic Cell biology, impacting clinical symptoms such as immune dysregulation, heightened infection risk and inappropriate chronic inflammation.

Therefore, in this review we have summarized the emerging roles of mitochondrial regulation in Dendritic Cell biology and discuss therapeutic opportunities to restore immune competence by targeting mitochondrial and redox pathways in settings of Dendritic Cell dysfunction. These insights aim to encourage further research into these topics and propose targeted metabolic reprogramming as a new therapeutic strategy for healthy ageing and chronic disease management.

## Graphical abstract

**Figure:**
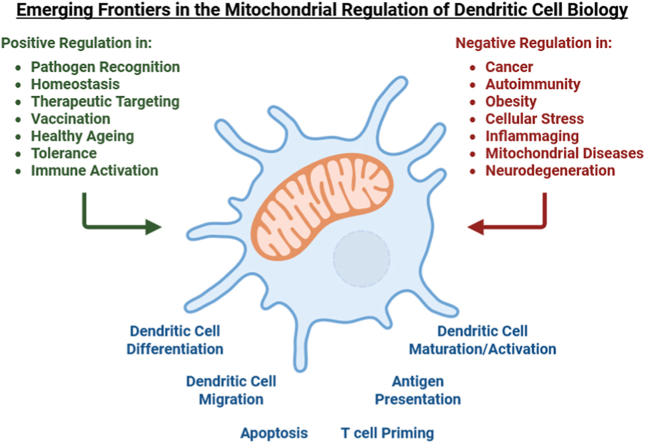

## 1 Introduction

Dendritic Cells (DC) are a heterogeneous population of professional antigen-presenting cells (APC) that serve as a critical interface between the innate and adaptive immune system. Their discovery over four decades ago highlighted their distinctive dendritic morphology and high capacity for antigen presentation [1,2]. Since then, DC have become a central focus of both basic and translational research due to their potent immunostimulatory capabilities and their pivotal role in orchestrating antigen-specific T cell responses.

DC are distributed throughout both lymphoid and non-lymphoid tissues and comprise multiple subsets with distinct developmental origins, phenotypes and functional specializations [3]. DC constantly develop within the bone marrow, with conventional dendritic cells (cDC) originating from myeloid precursors and plasmacytoid dendritic cells (pDC) differentiating from lymphoid precursors [4,5]. cDC can be further divided into cDC1, cDC2 and a more recently identified third DC3 subset [[6], [7], [8], [9]]. At the steady state these subsets display distinct transcriptional programs and phenotypic markers, as well as tissue-specific adaptations, to support uniquely tailored and context specific immune responses [10,11].

The unique functional roles of DC subsets are tightly controlled and are critical for the immune system's ability to detect and respond to pathogens. pDC are characterized by their capacity to produce large amounts of type I interferons, particularly in response to viral infections, and rapidly infiltrate tissues [12]. cDC1 excel at cross-presenting antigens and initiating cytotoxic CD8^+^ T cell responses within the draining lymph nodes (LN), making them central players in anti-viral and anti-tumor immunity, in addition to promoting Th1-type responses through IL-12 secretion [13]. cDC2, in contrast, demonstrate a broader versatility and support CD4 T cell differentiation in a context-dependent manner, driving Th2, Th17 and regulatory T cell (Treg) responses in a large variety of immunological contexts, while retaining the capacity to promote Th1 and CD8^+^ T cell responses under specific conditions [13]. The exact functionality of DC3 is still under investigation, but their role in promoting Th17 responses and supporting tissue-resident memory has been suggested [9]. To fulfill these specialized roles, previous reports have suggested that individual DC subsets rely on unique metabolic programs tailored to their specific immunological functions[[14], [15], [16]].

While only beginning to be understood, DC, like many other immune cells, undergo profound metabolic remodeling in response to environmental stimuli, a process that enables them to meet the energetic and biosynthetic demands of activation, antigen processing and cytokine production [15]. Intracellular metabolic pathways—such as glycolysis, oxidative phosphorylation (OXPHOS) and fatty acid oxidation (FAO)—are dynamically regulated during DC activation and perturbations in metabolic flux can markedly influence immune outcomes [16,17]. Furthermore, the metabolic regulation of DC can shape immunological outcomes in many situations, including infection, autoimmunity, tolerance, cancer, metabolic disorders and interactions with the gut microbiota [18].

Despite the growing recognition of metabolism as a critical regulator of immune cell fate and function, the specific contributions of mitochondria and redox biology to DC biology have received comparatively little attention. Mitochondria are highly dynamic, double-membrane-bound organelles that play essential roles not only in ATP production via the electron transport chain (ETC) and OXPHOS, but also in reactive oxygen species (ROS) generation, apoptosis and the synthesis of key metabolites including amino acids, lipids, nucleotides and heme [19]. Over 1500 proteins are required for mitochondrial function, 13 % of which are encoded by the mitochondrial genome, the remainder in the nucleus; requiring tight coordination between mitochondrial and nuclear transcriptional programs [20,21]. Several essential metabolic pathways, including OXPHOS, FAO, krebs cycle, urea cycle, amino acid metabolism and ascorbate metabolism either occur within mitochondria or are heavily dependent on mitochondrial enzymes and substrates [22,23]. Moreover, basic cellular functions are intimately linked to the number of cellular mitochondria, which is kept in the balance by mitochondrial fission, fusion and mitophagy events, which form a rheostat that constantly reacts to and adjusts cellular stress and its metabolic state [24].

In this review, we have therefore collected emerging knowledge on the role of mitochondrial activity in DC biology, with a particular focus on how mitochondrial pathways play a role in DC development, subset specialization and immune response modulation. By highlighting insights at the intersection of mitochondrial biology and immunology, we hope to provide a foundation for future studies and the development of new therapeutic strategies to target mitochondrial dysfunction.

## 2 Mitochondrial involvement in dendritic cell differentiation

Accumulating evidence suggests that mitochondria are directly and indirectly involved in the development and differentiation of DC, not only by providing the required energy for these biological processes, but also by acting as dynamic regulators of cellular fate and function.

The majority of studies investigating mitochondrial involvement in DC have been conducted using in vitro model systems (Table 1), including bone marrow-derived dendritic cells (BMDC) from mice and monocyte-derived dendritic cells (moDC) from human peripheral blood mononuclear cells (PBMC). These culture systems utilize different cytokines and growth factors to direct the differentiation of stem cells and progenitors towards specific DC subsets. FMS-like tyrosine kinase 3 ligand (FLT3L) drives the development of conventional DC subsets (cDC1, cDC2) and pDC-like cells [34,46], whereas granulocyte-macrophage colony-stimulating factor (GM-CSF) -with or without interleukin-4 (IL-4)- promotes the generation of moDC and macrophages [25,47,48]. However, in vitro DC models differ metabolically and functionally from bona fide DC subsets [14,18,25], thus care needs to be taken when extrapolating these findings into in vivo or clinical contexts.

**Table 1:** Emerging evidence of mitochondrial regulation in dendritic cell in vitro models.

Culture model	Cell origin	Culture conditions	Composition	Findings on mitochodrial regulation
**GM-CSF BMDC**	Murine bone marrow-derived progenitors	GM-CSF with or without IL-4 for 5–8 days	moDC-like and macrophage-like cells [25]	**Differentiation:** Increased mitochondrial fusion during BM progenitor differentiation [26]. **Activation:** Decline in OXPHOS following TLR engagement (LPS), attributed to the induction of iNOS and NO [27]. Treatment with IFN-β increased OXPHOS and enhanced expression of PGC-1β, supports mitochondrial biogenesis and respiratory capacity [28]. Mitochondrial membrane potential and spare respiratory capacity rose shortly after activation with LPS [29]. **Migration:** CCR7 ligation triggers mitochondrial fusion events [26,30]. **Antigen processing:** Mitochondria can translocate close to phagosomes/endosomes and supply local ATP to power dynein/kinesin motors (trafficking) to perinuclear lysosomes [31]. **T cell polarization**: FPR2-deficient DC show increased NO production, impaired mitochondrial respiration and defective production of Th17-skewing cytokines [32]. **Apoptosis:** Bax and Bak regulate Treg mediated apoptosis [33].
**FLT3L BMDC**	Murine bone marrow-derived progenitors	FLT3L for 9 days	Heterogeneous group of cDC- and pDC-like cells [34]	**Maturation:** TLR activation through CpG and Poly I:C increase the oxygen consumption rate through autocrine type 1 IFN signaling [35]. **T cell activation:** DC with higher oxidative metabolism have been shown to be more effective at cross-presenting antigens to CD8^+^ T cells and elicit enhanced cytotoxic T cell responses [36].
**iCD103+ BMDC**	Murine bone marrow-derived progenitors	FLT3L plus GM-CSF for 15–17 days	cDC1-like cells [37]	Not reported.
**Human moDC**	Human peripheral blood monocytes	GM-CSF plus IL-4 for 6–7 days	moDC-like cells [38]	**Differentiation:** Upregulation of mitochondrial function during the differentiation of monocytes into DC[[39], [40], [41]]. **Activation:** Activation through TLR7/8 using protamine-RNA complexes enhances OXPHOS in human moDC [42]. **Tolerance:** Cultures involving dexamethasone and/or vitamin D3 treatment exhibit elevated mitochondrial activity, including increased ROS production, spare respiratory capacity and assembled electron transport chain complexes [41,43]. AMPK activation renders human moDC tolerogenic through enhanced mitochondrial fission–dependent fatty acid oxidation [44]. **Apoptosis:** Loss of mitochondrial membrane potential and subsequent apoptosis after IL‐1α, IL‐6, TNF‐α, IL‐1β and prostaglandin E2 treatment [45].

Abbreviations: GM-CSF, granulocyte–macrophage colony-stimulating factor; cDC, conventional dendritic cell; FLT3L, FMS-like tyrosine kinase 3 ligand; moDC, monocyte-derived DC; pDC, plasmacytoid dendritic cell; LPS, lipopolysaccharide; Poly I:C, polyinosinic:polycytidylic acid; CpG, CpG (cytosine-phosphate-guanine) DNA motifs; OXPHOS, oxidative phosphorylation; iNOS, inducible nitric oxide synthase; NO, nitric oxide; PGC-1β, Peroxisome proliferator-activated receptor gamma coactivator-1β; TLR, toll-like receptor; FPR2, Formyl Peptide Receptor 2; AMPK, AMP-activated protein kinase.

**Fig. 1:**
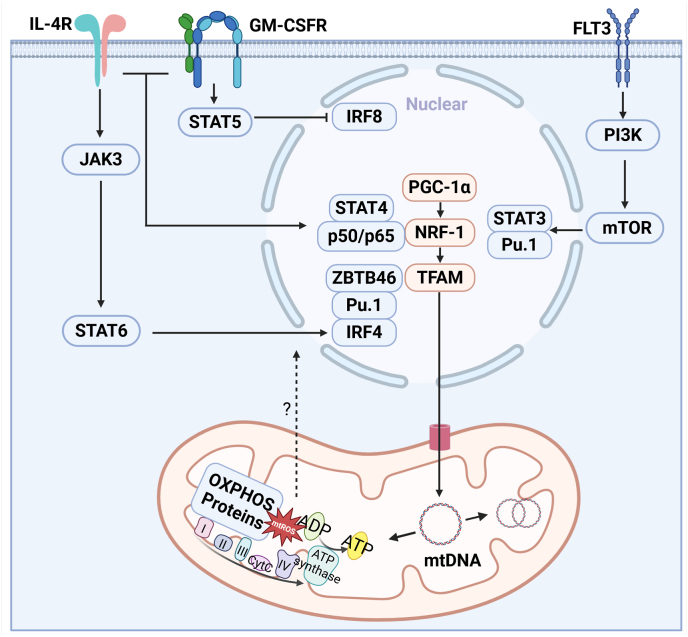
**Human moDC differentiation requires mitochondrial activity.** GM-CSF, IL-4 and FLT3L signaling affects DC differentiation through distinct molecular pathways. While IL-4 signaling induces STAT6 phosphoylation, GMCSF signals through STAT5 and FLT3L through mTOR. These transcription factors control different steps of DC differentiation both in murine and human cells (see Table 1). It has been shown that in human monocytes DC differentiation pathways also require mitochondrial regulation. Activation of PGC-1α and its downstream transcription factors NRF-1 and TFAM activate the expression and activity of mitochondrial respiratory complexes and increase mtDNA copy numbers. Thus, mitochondrial activation is likely required to provide the necessary energy and antioxidants for DC differentiation.

Human monocytes differentiated into DC using GM-CSF and IL-4 show pronounced upregulation of mitochondrial function, including increased mitochondrial DNA (mtDNA) copy number, elevated ATP production, enhanced expression and activity of respiratory complexes and elevated activity of the mitochondrial marker enzyme citrate synthase [39,40]. Over 6-fold and 4-fold differences in the enzymatic activity of Complex I and IV respectively were observed in GM-CSF + IL-4 cultured moDC compared to isolated monocytes [40]. Furthermore, cellular ATP content increased progressively throughout in vitro differentiation, from around 2 nmol/10^6^ cells at day 3 to over 12 nmol/10^6^ cells at day 6 and mtDNA copy numbers increased 3.5 fold [40]. Similarly another study reported that GM-CSF + IL-4 induce rapid reprogramming of glycolysis and transient co-activation of mitochondrial pathways in human monocytes using SCENITH analysis coupled with a multi-parametric FACS panel [41]. These changes are accompanied by increased mitochondrial mass and mitochondrial biogenesis, as TEM analysis showed 10–20 condensed mitochondria per cell section in GM-CSF + IL-4 DC compared to 2–3 in monocytes [40]. These increases were driven by the transcriptional coactivator peroxisome proliferator-activated receptor-γ coactivator-1α (PGC-1α), a master regulator of mitochondrial metabolism, promoting OXPHOS, FAO and ROS detoxification [49]. In the process of human monocytes differentiating into DC, PGC-1α is needed to trigger the subsequent activation of downstream transcription factors, such as nuclear respiratory factors (NRF)-1 and mitochondrial transcription factor A (TFAM), both essential for mitochondrial gene expression and replication [40] (Fig. 1). Disruption of mitochondrial function with rotenone, a complex I inhibitor, blocks this monocyte-to-DC transition, highlighting the dependence of mitochondrial respiration in DC differentiation [39].

Similar mitochondrial adaptations are observed in murine GM–CSF–derived BMDCs, where increased expression of levels of the mitochondrial fusion proteins Mitofusin 2 (MFN2) (13-fold) and mitochondrial dynamin like GTPase (OPA1) (8-fold) were observed at early stages of BM progenitor differentiation, suggesting an active remodeling of mitochondrial networks [26]. FLT3L-cultured BMDC exhibit marked transcriptomic differences (including distinct expression of the mitochondrial associated genes *Kmo*, *Ddx58* and *Slc7a11*) to GM–CSF–cultured cells [25]. While specific mitochondrial pathways have not been comprehensively characterized in FLT3L-derived DC, recent findings suggest that FAO plays a subset-specific role in DC development. Blocking FAO using etomoxir in FLT3L-driven BMDC cultures skews differentiation in favor of IRF4-dependent cDC2 at the expense of IRF8-dependent cDC1, further suggesting that metabolic programming influences lineage decisions [50]. Furthermore, inhibition of fatty acid synthesis significantly impairs moDC generation from human precursors and decreases DC numbers across lymphoid and non-lymphoid tissues in vivo, with FAO being essential for the expansion of CD11b^+^ DC in the context of inflammation [51].

Additional findings highlight possible interactions between known transcription factors important for DC differentiation and mitochondrial modulation. For example, the mTOR pathway (particularly mTORC1) is a crucial regulator of DC differentiation and regulator of mitochondrial activity and function [52]. Inhibition of mTORC1 via rapamycin impairs both cDC and pDC development in FLT3L- BMDC and reduces moDC survival in human DC cultures [53,54] (Fig. 1). Deletion of PTEN, a negative regulator of mTORC1, enhances DC outgrowth in vivo, while Raptor deletion (mTORC1 component) alters subset composition—elevating splenic cDC but reducing epidermal Langerhans cells [17]. Another example is MYCL, a member of transcription factors regulating nuclear and mitochondrial gene expression. During DC development, a switch in MYC paralog expression—from MYC to MYCL—occurs in cDC progenitors [55]. MYCL is essential for the normal development of CD103^+^ cDC and the normal function of CD8α^+^ cDC with MYCL-KO mice showing impaired T cell stimulation during infection [55]. Importantly, MYCL-deficient CD8α^+^ cDC display reduced expression of mitochondrial complex I genes, linking MYCL to mitochondrial respiratory and functional competence in cDC1 [56].

Together, these findings reveal a complex and nuanced role for mitochondrial metabolism in DC differentiation. In both human and murine culture models, mitochondrial biogenesis, OXPHOS activity, FAO and mitochondrial dynamics seem tightly coordinated to support the energetic and biosynthetic demands of DC development. Future work should aim to dissect DC subset-specific mitochondrial requirements, particularly in FLT3L-driven cDC differentiation and to validate these findings in in vivo settings.

## 3 Mitochondrial involvement in dendritic cell maturation and activation

DC maturation and activation are pivotal processes that transforms immature, antigen-capturing DC into potent initiators of adaptive immunity. DC maturation is triggered upon recognition of pathogen-associated molecular patterns (PAMPs) or danger-associated molecular patterns (DAMPs) via germline-encoded pattern recognition receptors (PRRs), such as Toll-like receptors (TLRs) [57]. Upon activation, DC increase surface expression of MHC class I and II, costimulatory molecules and the chemokine receptor CCR7, which enables their migration to or within secondary lymphoid organs where they initiate adaptive immune responses by presenting exogenous antigens to naïve T cells [58]. While traditionally believed to be exclusively governed by transcriptional and surface marker changes, growing evidence highlights the dynamic involvement of metabolic progresses, including their regulation by mitochondria.

**Fig. 2:**
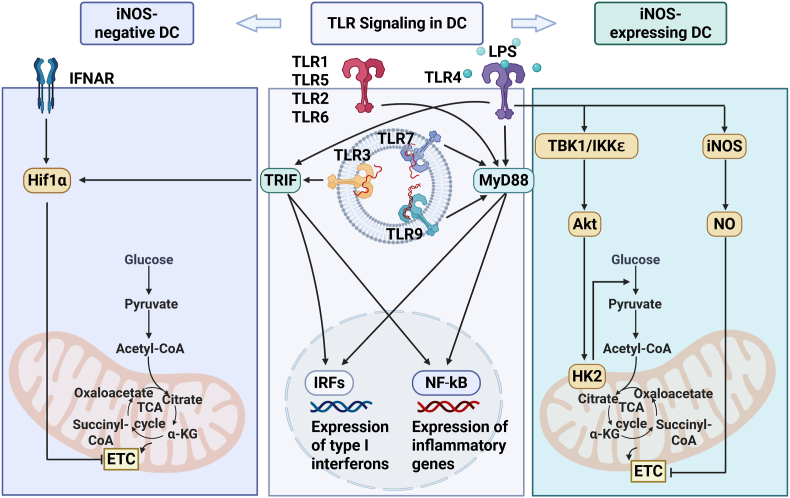
**Inhibition of immune suppressive OXPHOS signaling during DC activation.** Different groups of pathogen or danger associated molecules are recognized by specific TLRs leading to IRFs and NF-kB activation through TRIF or MyD88 signaling. While IRF and NF-kB activation leads to the expression of type I interferons and inflammatory cytokines, DC activation is also accompanied by an increase in glycolysis and an inhibition of OXPHOS. Signaling via TBK1, IKKɛ and Akt promote the association of the glycolytic enzyme HK2 with mitochondria and is essential for the TLR-induced increase in glycolysis after LPS stimulation. While not yet studied for all TLRs, different mechanisms have been identified leading to the concurrent inhibition of OXPHOS. In iNOS-negative DC, e.g. murine cDC, the inhibition of the Electron Transport Chain (ETC) and OXPHOS is regulated by IFNAR signaling and HIF1α expression induced by TLR stimulation. In iNOS-expressing human moDC TLR agonists instead induce the expression of iNOS, leading to the inhibition of OXPHOS by NO.

Upon stimulation with TLR agonists, DC undergo rapid metabolic remodeling and changes in their redox biology. This includes increased glucose uptake and lactate production, hallmarks of a cellular glycolytic shift akin to the Warburg effect observed in tumor cells [27,59,60]. Although glycolysis dominates the metabolic landscape during DC activation, mitochondrial function plays complex and sometimes paradoxical roles throughout this process. In vitro models show that initial DC activation is often accompanied by a decline in OXPHOS following TLR engagement, determined by extracellular flux analysis (measurements of mitochondrial oxygen consumption rate), particularly through TLR4 stimulation with LPS [27,60]. This decline is, in part, attributed to the induction of inducible nitric oxide synthase (iNOS), which leads to the generation of nitric oxide (NO) [27]. In moDC NO inhibits mitochondrial respiration by targeting complex IV, thereby enforcing a metabolic switch to glycolysis to maintain ATP levels under inflammatory conditions [27](Fig. 2). In vivo, iNOS expression and NO production are largely restricted to moDC and are absent in cDC subsets within secondary lymphoid tissues [61], suggesting subset-specific mitochondrial adaptations.

In murine iNOS-negative CD11c+ MHC II+ cDC, it has instead been shown that type I interferon signaling dominates the transcriptional response to TLR agonists [60] (Fig. 2). Using WT and IFNAR−/− DC mixed bone marrow-chimeric mice, it was observed that IFNAR signaling leads to upregulation of hypoxia-inducible factor 1α (Hif1α), a master regulator of glycolysis, which suppresses mitochondrial function and subsequent ROS production [60].

While TLR activation tends to suppress OXPHOS signaling, treatment with IFN-β led to an increase of OXPHOS and enhanced expression of PGC-1β, a known transcriptional coactivator that supports mitochondrial biogenesis and respiratory capacity, in murine GM-CSF cultured BMDC, while LPS-treated controls upregulated glycolysis [28]. Similarly, in human moDC cultures, TLR4 stimulation with LPS induced a glycolytic switch, whereas activation through TLR7/8 using protamine-RNA complexes enhances OXPHOS [42]. Mechanistically, OXPHOS upregulation was mediated through PTEN-induced putative kinase 1 (PINK1)-dependent phosphorylation of branched-chain ketoacid dehydrogenase E1α (BCKDE1α), promoting FAO and subsequent mitochondrial activation, ultimately supporting moDC maturation [42]. These findings suggest that the metabolic outcome of DC activation depends on the nature of the activating stimulus and the downstream signaling cascade.

Furthermore, real-time fluctuations in the rate of extracellular acidification (ECAR), as well as the mitochondrial rate of oxygen consumption (OCR) have been studied in DC. After activation with LPS, the OCR remained stable, while ECAR increased rapidly within 125 min in murine BMDC, confirming previous observations of a glycolytic switch [29](Fig. 2). Additionally, mitochondrial membrane potential and spare respiratory capacity rose shortly after activation, indicative of enhanced mitochondrial activity [29].

In contrast to cDC, which exhibit a greater dependence on glycolysis for their activation, the activation of pDC is predominantly reliant on OXPHOS, which might be related to the functional difference between cDC and pDC. pDC poorly present exogenous antigens, as they are unable to take up antigens by phagocytosis or micropinocytosis and do not accumulate long-lived peptide–MHC class II complexes on their cell surface after activation [62]. Therefore the rapid production of type I interferons is considered the major immunological function of pDC.

In response to TLR activation with CpG, CD11c^int^ Siglec H^+^ B220^+^ pDC from FLT3L BMDC cultures produced type I interferons, which, through an autocrine type I interferon receptor-dependent pathway, induced increased FAO and OXPHOS [35]. In isolated human BDCA2^+^ CD123^+^ pDC similar observations were made upon TLR7 and TLR9 activation by Influenza A or Herpes Simplex Virus, respectively and TLR7/8-stimulation by protamine-RNA increasing OXPHOS, mitochondrial content and intracellular glutamine in an autophagy-dependent manner [63,64]. Interestingly, increased mitochondrial activity has been linked to different immune functions in pDC, as OX40^+^ pDC (which showed higher mitochondrial volumes and OXPHOS) were superior to both OX40^lo^ and OX40^–^ pDC in boosting mDC priming of CD8^+^ T cells [65]. Mitochondrial superoxide production also has an impact on pDC activation, as mtROS induction with Antimycin-A decreases CpG-induced expression of type I interferons both at mRNA and protein level in the human GEN2.2 pDC cell line [66]. Therefore, ROS levels might also play a role in regulating the early production of type I interferon in pDC upon TLR activation.

Another possibility of the reliance on OXPHOS by pDC after activation might be a decreased ability to take up or process glucose. 2-NBDG levels (a fluorescent tracer used for monitoring glucose uptake in living cells) was unchanged in freshly isolated human pDC after pRNA-stimulation, while being significantly increased CD1c + mDC [63]. Our own analysis of published single-cell RNA-seq datasets [9,67,68] also show that key enzymes of glycolysis such as HK2 and PFKFB3 are expressed at lower levels in pDC compared to cDC or activated DC. In addition differences in anabolic metabolism or antagonizing mTORC1 and AMPK signaling might lead to different mitochondrial states in pDC and cDC, as reviewed in Ref. [69].

Collectively, these findings highlight the context-dependent and subset-specific roles of mitochondria and redox biology in DC maturation and activation, with distinct pathways being involved in moDC, cDC and pDC activation. Further experiments are necessary to define the full picture of mitochondrial control in vivo and under complex disease settings, providing exciting avenues for future research activities.

## 4 Role of mitochondria in dendritic cell migration, antigen presentation and T cell activation

Beyond their role in DC development and maturation, mitochondria are emerging as regulators of several functional hallmarks of DC immunobiology, including migration, antigen presentation and T cell priming. Initial findings suggest that these processes require substantial metabolic adaptation and are intimately linked to mitochondrial bioenergetics, dynamics, signaling and redox biology.

While DC arrive to lymphoid and non-lymphoid organs via the blood stream, a substantial and immunologically highly relevant proportion of DC subsequently migrate from peripheral tissues to the draining LN upon activation. This migration is mediated by CCL21 chemotaxis gradients expressed by lymphatic endothelial cells in tissues and require the expression of CCR7 by activated DC [70]. Thus, DC migration and activation are intimately linked [71,72]. Within lymphoid organs, both tissue-derived and blood-derived DC can also migrate from the subcapsular sinus of the LN to the paracortex or in case of the spleen (which only contains blood-derived DC) from the marginal zone to the white pulp via CCL19/CCR7 recognition [73]. While migration events within LN and the spleen are less studied from a metabolic perspective, several findings highlight that CCR7 activation is metabolically demanding and depends on mitochondrial activity. Specifically, mitochondrial fusion events have been observed via transmission electron microscopy in mDCs stimulated with CCR7L [30]. CCR7-activation led to increased mitochondrial oxidative respiration and mitochondrial membrane potential measured by oxygen consumption rate analysis and TMRE staining respectively [30]. This increase was mediated by Farnesyl pyrophosphate (FPP), one of the key enzymes involved in the mevalonate pathway, leading to RhoA geranylgeranylation and ER stress [30,74]. Furthermore, other studies have shown that mitochondrial fusion-related proteins, such as Mfn2 and OPA1, regulate the expression of CCR7 and interact with cytoskeleton signaling [26], altogether indicating that mitochondrial dynamic and function support the energy-intensive process of DC migration. However, differences might exist between strongly and weakly activated DC, as weakly activated DC have been shown to lack long-term HIF-1α-dependent glycolytic reprogramming and retain mitochondrial oxidative metabolism [75].

**Fig. 3:**
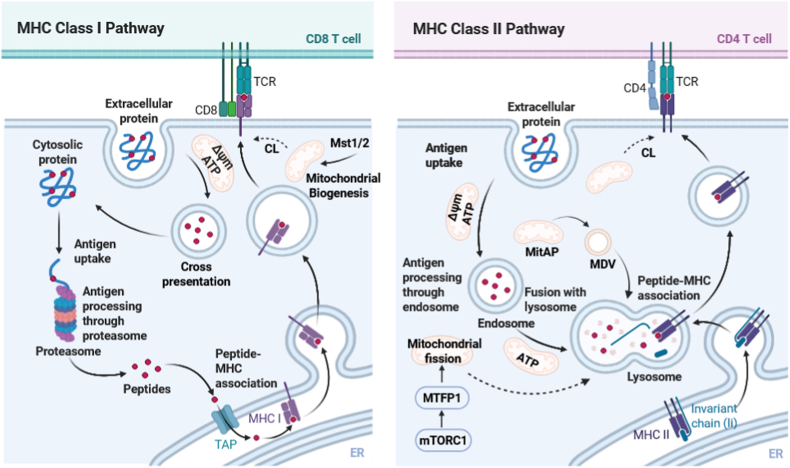
**Mitochondrial involvement in antigen processing and presentation.** Two classes of MHC exist, which differ in their peptide loading and immune cell interaction repertoire. MHC I peptides are mainly derived from cytosolic antigens and presented to CD8 T cells. While these mechanisms have evolved to recognize virally infected cells and tumors, they can also be used by DC to present extracellular antigens in a process called cross-presentation. MHC II loading is specialized to process and present extracellular antigens to elicit CD4 T cell responses. Mitochondria contribute to these processes in several stages. High mitochondrial membrane potential (Δψm) and ATP levels correlate with robust antigen internalization. Mitochondria also contribute to the maturation of antigen-containing endosomes and translocate close to phagosomes/endosomes and supply local ATP to power dynein/kinesin trafficking motors to reach perinuclear lysosomes, supporting the trafficking and loading of peptide–MHC complexes. Hippo pathway kinases Mst1 and Mst2 modify fusion regulators OPA1 and DRP1, leading to increased bioenergetic mitochondrial output and robust CD8^+^ cytotoxic T lymphocyte responses. Similarly, mTORC1 stimulates translation of MTFP1 controlling mitochondrial fission, increasing lysosomal content and energetic output. Cardiolipin (CL) expression, induced by mitochondrial signaling, has also been shown to improve T cell priming function. Novel mechanisms that suggest a direct involvement of mitochondria in antigen presentation (e.g. Mitochondrial antigen presentation and mitochondria derived vesicles) have also been proposed, but require further investigation.

Efficient antigen uptake by DC involving phagocytosis and endocytosis, is also closely tied to mitochondrial function (Fig. 3). High mitochondrial membrane potential (Δψm) and ATP levels correlate with robust antigen internalization of splenic DC, while impaired mitochondrial activity results in diminished phagocytic capacity [76]. Mitochondria may further contribute to the maturation of antigen-containing endosomes, supporting the trafficking and loading of peptide–MHC complexes [31]. Mechanistically, mitochondria can translocate close to phagosomes/endosomes and supply local ATP to power dynein/kinesin trafficking motors to reach perinuclear lysosomes. This has been experimentally validated in GM-CSF cultured BMDC by inducing Δψm impairment, which interfered with LPS-mediated antigenic-endosome transport to the perinuclear zone [31]. The involvement of mTOR signaling has also been suggested to influence lysosome biology. It is well established that the nutrient-sensing mechanistic/mammalian target of rapamycin complex 1 (mTORC1) stimulates the translation of the mitochondrial fission process 1 (MTFP1) to control mitochondrial fission and apoptosis in many cell types and cell lines [77]. Expression of MTFP1 is coupled to pro-fission phosphorylation and mitochondrial recruitment of the fission GTPase dynamin-related protein 1 (DRP1) [77]. Yet, in keratinocytes it has also been shown that constitutive mTORC1 activation can increase lysosomal content via upregulated expression and activity of the microphthalmia family of transcription factors (MiT/TFEs) [78], which might represent a new mechanism of how mTOR signaling is involved in lysosome biogenesis.

Enhanced mitochondrial activity also plays a role in the T cell activation capacity of DC, particularly in stimulating robust CD8^+^ cytotoxic T lymphocyte (CTL) responses. DC1 show higher oxidative metabolism and have consistently been shown to be more effective at cross-presenting antigens to CD8^+^ T cells and to elicit enhanced CTL responses to viruses, bacteria and tumors [36]. These observations have been supported by studies in DC-T cell co-cultures, which show that treatment of metformin (an inhibitor of mitochondrial complex I activity) markedly impairs the ability of DC1 to prime OT-I T cells [36]. Mechanistically, Hippo pathway kinases Mst1 and Mst2 were identified as important regulators of mitochondrial morphology and function during DC1-mediated T cell priming. Mst1/2-deficient DC1 exhibit reduced expression of the mitochondrial fusion regulator OPA1 and reduced phosphorylation of the fission protein DRP1 at Ser637, leading to reduced bioenergetic mitochondrial output [36].

Similarly, GM-CSF BMDC which contain unstable mitochondria (experimentally induced by carbon monoxide signaling in vitro) displayed reduced mitochondrial membrane potential, ATP production and less effective T cell priming potential [31]. Furthermore, GMCSF + IL-4 BMDC treated with PEG–COOH–coated Fe_3_O_4_ nanoparticles exhibit mitochondrial instability, loss of Δψm and increased mitochondrial fusion resulting in reduced levels of dextran uptake and CD80, CD86 and CCR7 expression [79].

However, mitochondrial dysfunction can have paradoxical effects on T cell priming. In the context of lung tumors, deletion of TFAM—a key transcription factor required for mitochondrial DNA replication and maintenance—leads to mitochondrial dysregulation, including mitochondria swelling, cristae loss, ROS accumulation and mtDNA cytosolic leakage [80]. These mitochondrial defects however activate the cGAS-STING pathway, improve antigen presentation and CD8^+^ T cell proliferation within lung tumors and overcome the suppressive tumor microenvironment [80]. However in hepatocellular carcinoma, tumor-secreted oncofetal protein α-fetoprotein (AFP) reduces mitochondrial respiration and ATP production in DC [81]. As a consequence, DC exposed to AFP fail to effectively prime antigen-specific CD8^+^ T cells, resulting in diminished TNF-α secretion and reduced CD69 expression on T cells [81]. Furthermore, glucose has been shown to repress DC-induced T cell responses and is also required for the inactivation of OXPHOS through mTORC1, HIF1a, iNOS and NO [82](Fig. 3).

During T cell priming, mitochondria dynamically cluster at the immunological synapse, the contact site between DC and T cells. This localization facilitates ROS production (defined by increased DHE fluorescence), which is required to stabilize the synapse and support sustained T cell activation [83]. In addition, mitochondrial activity modulates the secretion of T cell-polarizing cytokines in DC. For example, formyl peptide receptor 2 (FPR2) influences DC metabolism and cytokine output, as FPR2-deficient DC show increased NO production, impaired mitochondrial respiration and defective production of Th17-skewing cytokines, linking mitochondrial function to T helper cell polarization [32].

Mitochondrial regulation is also critical in human tolerogenic DC (TolDC), which have been linked to immune tolerance and the suppression of effector T cell proliferation. TolDC are characterized by a catabolic metabolic phenotype, encompassing increased oxidative phosphorylation, fatty acid metabolism and glycolysis and primarily generated by culturing moDC with dexamethasone and/or vitamin D_3_ [43] (Table 1). TolDC exhibit elevated mitochondrial activity, including increased levels of ROS production, spare respiratory capacity and assembled electron transport chain complexes [43]. While it is still under debate if TolDC constitute a distinct DC subset, functional phenotype or culture specific adaptation, enhanced mitochondrial fission–dependent fatty acid oxidation and AMPK-induced RALDH activity have been observed in TolDC cultures, while basal mitochondrial respiration, ATP production, spare respiratory capacity, mitochondrial mass or membrane potential remained unaffected [44]. A proportional shift from glucose dependence to FAAO utilization in human TolDC generated with 1α,25-dihydroxyvitamin D_3_ (VitD_3_) has also been observed on a single cell level using SCENITH measurements [41], suggesting a potential role of OXPHOS in TolDC. Although the metabolic distinctions between TolDC and immunogenic DCs are well documented, the precise links between mitochondrial metabolism and tolDC function, which is controlled by STAT3, AHR, SOCS2 and other transcription factors, yet remains to be defined [84].

Thus, mitochondrial metabolism is closely linked to CCR7 dependent DC migration, antigen processing and presentation and T cell activation, as well as tolerance. Despite increasing recognition of mitochondria as central hubs in coordinating these immunological functions, the precise molecular mechanisms are still incompletely understood in many instances. Recent advances in high-resolution omics technologies, such as single-cell transcriptomics, spatial metabolomics, ribosome profiling and mitochondrial proteomics, are poised to close these knowledge gaps and advance our understanding of how mitochondrial processes regulate DC function and T cell priming [85].

## 5 Role of mitochondria in dendritic cell apoptosis

Apoptosis, or programmed cell death, is a tightly regulated process essential for the maintenance of homeostasis. It also plays a critical role in controlling the initiation, magnitude, duration and resolution of immune responses [86]. In this context DC apoptosis ensures the proper turnover of these potent APC limiting excessive immune activation that could otherwise lead to autoimmunity or chronic inflammation.

Two principal pathways control apoptosis in eukaryotic cells: the extrinsic and intrinsic pathway. The extrinsic pathway is initiated by the binding of death-inducing ligands—such as Fas ligand (FasL) or tumor necrosis factor (TNF)—to their respective death receptors on the cell surface. This interaction leads to the assembly of the death-inducing signaling complex (DISC), which in turn activates initiator caspases, predominantly caspase-8 and, in humans, caspase-10 [87].Once activated, these caspases initiate a downstream proteolytic cascade that culminates in cell death [87]. On the other hand, the intrinsic pathway, also known as the mitochondrial pathway, is triggered by intracellular stress signals such as DNA damage, oxidative stress or growth factor withdrawal [88]. Central to this pathway is the process of mitochondrial outer membrane permeabilization (MOMP), a critical point of no return. MOMP allows for the release of key pro-apoptotic factors, including cytochrome *c*, from the mitochondrial intermembrane space into the cytosol [89]. Once in the cytosol, cytochrome *c* binds to apoptotic protease activating factor-1 (APAF1), promoting the formation of the apoptosome—a multiprotein complex that recruits and activates caspase-9, the initiator caspase of the intrinsic pathway [90,91]. Caspase-9 then activates downstream effector caspases, such as caspase-3 and caspase-7, ultimately executing the cell death program. Regulation of MOMP is primarily mediated by the Bcl-2 family of proteins, which includes both pro-apoptotic members (e.g. Bax, Bak, and Bim) and anti-apoptotic members (e.g. Bcl-2 and Bcl-xL) [92]. These proteins collectively govern the integrity of the mitochondrial membrane and the formation of the mitochondrial permeability transition pore (mPTP).

**Fig. 4:**
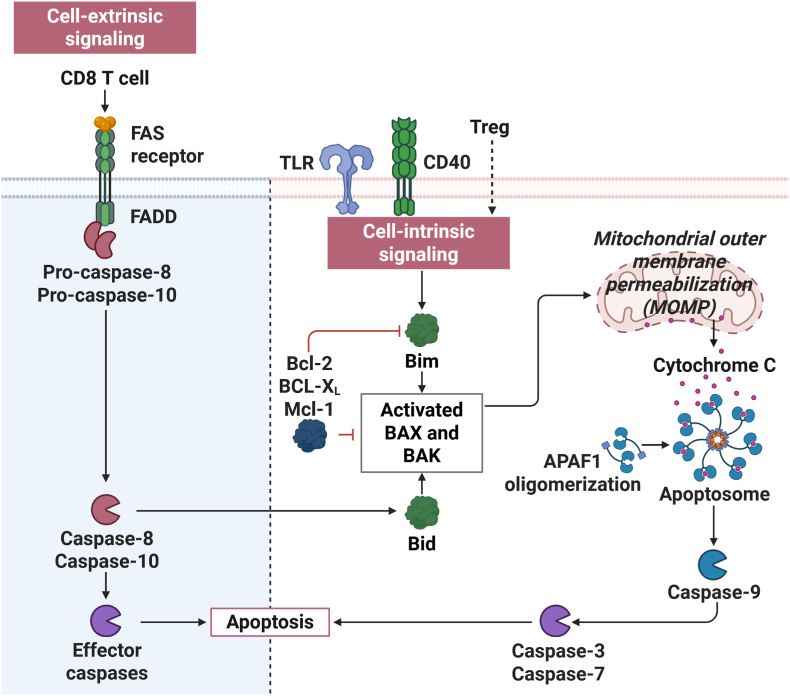
**Apoptosis signaling in DC and the involvement of mitochondria.** In DC, cell-extrinsic apoptosis can be triggered by CD8^+^ T cells through Fas-FASL signaling, leading to the activation of caspase-8 and caspase-10 and the induction of mitochondrial degradation and apoptosis. Cell-intrinsic signaling can be induced by PRR signaling or Tregs and lead to the activation of Bcl-2 family members resulting in mitochondrial outer membrane permeabilization, the activation of the Apoptosome and Apoptosis. Controlled DC apoptosis is one of the mechanisms of how misrepresentation of the actively presented antigen pool or over-activation of the immune system are thought to be regulated.

DC, despite their central role in immune activation, are inherently short-lived, a feature attributed to their high sensitivity to apoptotic signals. Following the priming of T cells, DC are often eliminated within secondary lymphoid tissues through both extrinsic and intrinsic pathways [87,88](Fig. 4). For example, CD8^+^ cytotoxic T cells can induce DC apoptosis through the extrinsic pathway by engaging Fas (CD95), leading to DISC formation and caspase-8 activation [93]. This process ensures the removal of antigen-presenting DC after their role in T cell priming is complete, thereby preventing prolonged stimulation. Regulatory T cells on the other hand promote DC apoptosis in the periphery through the intrinsic pathway. Tregs induce mitochondrial dysfunction by activating pro-apoptotic proteins Bax and Bak, which facilitate MOMP and the release of apoptogenic factors from mitochondria, revealed by experiments that showed that Bax^−/−^ Bak^−/−^DC were more resistant to killing by Tregs [33]. The activity of these proteins is tightly regulated by the broader Bcl-2 family network and is crucial not only for Treg-mediated immunosuppression but also for maintaining the natural turnover of DC. Indeed, the short half-life of DC has been linked to several signals that can initiate the intrinsic apoptotic pathway[[94], [95], [96]]. Emerging evidence suggests that apoptotic regulation in DC is for example directly regulated by PRR signaling. For instance stimulation of CD40 or TLRs in murine DC induced the expression of Bim, a pro-apoptotic member of the Bcl-2 family, which led to spontaneous cell death after DC activation both shown in vitro and in vivo [96]. This mechanism may act as a feedback loop to prevent overstimulation of the immune system. Interestingly, another study also points to the possibility that engagement of MHC II molecules, independent of traditional death receptor signaling, may itself lead to a loss of mitochondrial membrane potential and subsequent apoptosis [45](Fig. 4). These findings suggest that the antigen-presentation machinery itself may limit the duration of DC activity by triggering cell death, thus limiting over-stimulation.

Together, these findings highlight the multifaceted role of mitochondria in regulating DC lifespan and function. Mitochondrial signaling not only dictates cell survival or cell death, but also serves as a crucial checkpoint in maintaining immune equilibrium. Future investigations into DC subsets and comparisons with other short-lived immune cells, like monocytes and neutrophils, will be instrumental in unraveling the intricate network between metabolic, apoptotic and immunological signals governing DC biology.

## 6 Therapeutic strategies to counteract the decline in mitochondrial function in ageing and other diseases

In previous sections we have highlighted how mitochondrial signaling is emerging as an important regulator of DC biology, including their differentiation, activation, migration, T cell priming potential and lifespan. Considering this important role, it follows that mitochondrial dysfunction, which is prevalent in various diseases, could directly limit DC functionality and thereby disrupt immune homeostasis. While mitochondrial dysregulation in DC within the tumor microenvironment has been extensively reviewed [97,98], less attention has been given to conditions with systemic mitochondrial dysfunction, such as aging and mitochondrial diseases (MD).

Aging is closely associated with a decline in mitochondrial function and linked to the free radical theory of aging that connects increased production of mitochondrial ROS to cellular aging and organismal senescence [99,100]. While cellular senescence is predominantly associated with decreased cellular functionality mitochondrial dysfunction has also been associated with “inflammaging”, a chronic, low-grade inflammatory state characterized by sustained innate immune activation and reduced flexibility of the immune response [101,102].

The relationship between aging and DC dysfunction has been examined in several studies [103]. DC derived from aged animal models exhibit marked mitochondrial dysfunction, which manifests in decreased mitochondrial membrane potential, impaired ATP turnover, reduced coupling efficiency, diminished baseline oxidative phosphorylation, increased proton leak and elevated mitochondrial ROS production [76]. This dysregulation is linked to decreased DC functionality in vitro, particularly a reduced phagocytic capacity, a less efficient ability to cross-present cell-associated antigens and less efficient cross-priming of CD8^+^ T cells [76]. Mitochondrial dysfunction can also induce senescence in DC, which is characterized by irreversible cell-cycle arrest and the acquisition of a senescence-associated secretory phenotype (SASP) [104]. Mechanistically, ATM, Akt and mTORC1 signals drive PGC-1β-dependent mitochondrial biogenesis, contributing to ROS-mediated activation of the DNA damage response and cell cycle arrest [105]. Analysis of RNA-seq data from circulatory CD1c + DC from aged and young individuals also show that genes linked to the mitochondrial electron transport chain are downregulated in DC from aged individuals, suggesting mitochondrial dysfunction and a reduced energy metabolism in DC during ageing [106]. Increased cellular senescence has been associated with decreased functionality in murine studies, where APC from the peritoneal cavity of old mice induced lower lymphocyte cytotoxicity and IFNγ-producing T cells in comparison with APC from young animals, with lower levels of activation markers and CCR7 being observed [107]. Similarly, BMDC cultured from aged bone marrow cells were less effective in stimulating OVA-specific CD4^+^ T cell proliferation and showed less effective DC migration after adoptive transfer into young mice [108]. While the changed mitochondrial biology of DC during ageing is just beginning to be unraveled, studies from other myeloid cell types, such as monocytes, macrophages and neutrophils, suggest profound changes [109]. In human monocytes it has been shown that the mitochondrial respiratory capacity is significantly impaired and reduced by almost half, suggesting reduced mitochondrial fitness [110]. Mouse studies obtained similar findings, showing impaired oxidative phosphorylation and increased glycolytic ATP production in macrophages and neutrophils [111,112], which strongly links age associated mitochondrial dysfunction with negative impacts on myeloid cell functionality [113].

Another important group of diseases associated with systemic mitochondrial dysfunction are Mitochondrial diseases (MD). They comprise a clinically heterogeneous group of genetic disorders caused by mutations in nuclear or mitochondrial DNA that affect structural or functional mitochondrial proteins leading to defects in OXPHOS [20,114]. The prevalence of MD is estimated at 1 in 5000 births, yet symptoms can occur across all age groups and can vary widely depending on the gene and organs involved, often resulting in relentless progression, high morbidity and mortality [114]. A strong link between MD and reduced immune functions has been established. Infections constitute a major cause of morbidity and mortality in MD patients, with many reporting recurrent infections and delayed recovery following infectious episodes. Approximately 79 % of MD patients report prolonged convalescence, with recurrent sinus and fungal infections being dominant [115]. A second study documented that 42 % of patients with MD suffered serious or recurrent infections and a significant subset experienced systemic inflammatory response syndrome (SIRS) [116]. These clinical findings align with epidemiological data showing that sepsis and pneumonia are leading causes of death in MD [117].

Innate immune responses and DC-driven adaptive immunity are essential for controlling infections, implicating DC dysfunction as a potential factor in MD-associated immune dysregulation. Despite the lack of direct studies investigating DC function in MD, impaired immune competence remains a recognized contributor of acute clinical deterioration in MD [118]. Gene set enrichment analyses of transcriptomic data from MD patients reveal positive enrichment of DC and monocyte gene signatures, whereas T and B cell gene sets are downregulated [119]. This transcriptional profile correlates with heightened inflammatory signaling pathways, including type I interferon, interleukin-1β and antiviral responses, indicating dysregulated immune activation of APC [119]. Furthermore, infections and exaggerated TLR signaling may exacerbate disease progression. For example, 61 % of acute exacerbations and hospitalizations in Leigh syndrome—a severe mitochondrial encephalopathy—are infection-related [120]. It might even be the case that a feedback loop between recurrent antigenic stimulation or chronic inflammatory signaling and immune cell exhaustion exists in MD, a phenomenon documented in several examples of T cell and pDC over-activation [121,122], leading to a progressive loss of immune cell function and sustained expression of inhibitory receptors, further compromising host defense.

Therapeutically, these insights highlight the potential of targeting mitochondrial signaling pathways in innate immune cells, including DC, to restore immune competence in MD and mitigate age-associated immune decline. Approaches to normalize mitochondrial bioenergetics, reduce oxidative stress or modulate mitochondrial apoptosis pathways could enhance DC functions and improve host defense. Considering the role of OXPHOS in CD8^+^ T cell induction, different OXPHOS substrates might for example serve as additives for therapeutic formulations. Indeed, studies showed that high levels of endogenous omega-3 fatty acids promote DC antigen presentation and improve DC–based cancer vaccine efficacy in mice [123] and lead to increased OXPHOS in in vitro cultured PBMC and macrophages [124].

Other advances in mitochondrial therapeutics, including mitochondrial replacement therapies, antioxidants targeting to mitochondria and metabolic modulators, could also offer promising avenues for intervention [125,126]. Moreover, modulation of mitochondrial dynamics and mitophagy have emerged as strategies to maintain mitochondrial quality, potentially preserving DC functionality under stress conditions [127]. Delivery mechanisms to target DC specifically or APC in general might involve the conjugation to antibodies or ligands that recognize DC-restricted surface receptors or the use targeted nanocarriers [128]. Alternatively, DC-specific promoters (e.g., CD11c or CLEC9A/cDC1 promoters) can be employed in gene delivery systems—such as viral vectors or DNA plasmids—to drive selective expression of mitochondrial modulators in DC [129]. While these approaches still need to be tested experimentally, ex vivo loading of DC with mitochondria-targeted nanoparticles has already been attempted and shown promise in enhancing specific DC functions and immune responses in vitro [130].

In summary, mitochondrial dysfunction in DC might contribute to systemic immune defects in aging and MD, potentially accelerating immunosenescence and infection susceptibility. More research is needed to fully understand the molecular details of the mitochondrial-immune crosstalk in DC to fully develop innovative therapies aimed at restoring immune homeostasis through mitochondrial reprogramming.

## 7 Conclusions

As we have reviewed above, mitochondria are emerging as important regulators of DC biology, with numerous studies demonstrating a strong link between mitochondrial activity and DC development and immune function. Yet, significant gaps remain in our understanding—particularly regarding how mitochondrial programs operate in vivo and how they influence key processes such as DC migration and the induction of immune tolerance.

While in vitro studies have illuminated many aspects of mitochondrial control in DC differentiation, it remains unclear whether these mechanisms can faithfully be extrapolated to different DC subsets or from in vitro to in vivo systems. Given the divergent mitochondrial involvement in DC and pDC activation it is likely that additional research is necessary to define the specific roles mitochondria play in different DC subsets and disease contexts. Other areas of research involve the role of mitochondria in DC migration, a process critical for tissue surveillance and T cell priming. Investigating how mitochondrial dynamics intersect with cytoskeletal regulation and chemotactic responses will be key to addressing these questions, as studies investigating actual migration events are still lacking. Furthermore, clarifying the temporal contributions of mitochondrial versus glycolytic metabolism during DC activation is essential for a more nuanced understanding of DC function and effective targeting in dysregulated contexts.

An additional challenge in the field of DC biology lies in the variability of experimental models. DC generated in vitro using GM-CSF, IL-4 or Flt3L, or purified ex vivo from tissues or blood, often display divergent functional and metabolic profiles in response to identical stimuli (Table 1), complicating cross-study comparisons. To gain physiologically relevant insights it is imperative that mitochondrial function be investigated within DC in their native tissue environments using transgenic or single-cell omics approaches. Both biased and unbiased metabolomics, ranging from isotope tracing to metabolic flux analysis and spatial metabolite distribution are gaining traction in several fields of research and can be readily applied to study mitochondrial functions in DC [131]. Single-cell omics approaches, using transcriptomic, epigenetic and even proteomic readouts are becoming better and better in defining metabolic and mitochondrial states offering the resolution needed to capture dynamic and cell type and subset specific mitochondrial programs[[132], [133], [134]].

Closing the gap of how mitochondrial regulation controls DC biology in vivo will be critical for translating these insights into strategies that restore immune homeostasis across health and disease to benefit healthy ageing, mitochondrial diseases, but also cancer, autoimmunity and vaccination.

## CRediT authorship contribution statement

**B. Chen:** Funding acquisition, Investigation, Methodology, Resources, Visualization, Writing – original draft, Writing – review & editing. **J.U. Mayer:** Conceptualization, Funding acquisition, Investigation, Project administration, Resources, Supervision, Validation, Writing – original draft, Writing – review & editing.

## Declaration of competing interest

none.

## Data Availability

No data was used for the research described in the article.
